# An SVM-Based Solution for Fault Detection in Wind Turbines

**DOI:** 10.3390/s150305627

**Published:** 2015-03-09

**Authors:** Pedro Santos, Luisa F. Villa, Aníbal Reñones, Andres Bustillo, Jesús Maudes

**Affiliations:** 1 Department of Civil Engineering, University of Burgos, C/ Francisco de Vitoria s/n, Burgos 09006, Spain; E-Mails: psgonzalez@ubu.es (P.S.); jmaudes@ubu.es (J.M.); 2 CARTIF Foundation, Parque Tecnológico de Boecillo, Boecillo 47151, Spain; E-Mails: lvillamo@eafit.edu.co (L.F.V.); aniren@cartif.es (A.R.)

**Keywords:** fault diagnosis, neural networks, support vector machines, wind turbines

## Abstract

Research into fault diagnosis in machines with a wide range of variable loads and speeds, such as wind turbines, is of great industrial interest. Analysis of the power signals emitted by wind turbines for the diagnosis of mechanical faults in their mechanical transmission chain is insufficient. A successful diagnosis requires the inclusion of accelerometers to evaluate vibrations. This work presents a multi-sensory system for fault diagnosis in wind turbines, combined with a data-mining solution for the classification of the operational state of the turbine. The selected sensors are accelerometers, in which vibration signals are processed using angular resampling techniques and electrical, torque and speed measurements. Support vector machines (SVMs) are selected for the classification task, including two traditional and two promising new kernels. This multi-sensory system has been validated on a test-bed that simulates the real conditions of wind turbines with two fault typologies: misalignment and imbalance. Comparison of SVM performance with the results of artificial neural networks (ANNs) shows that linear kernel SVM outperforms other kernels and ANNs in terms of accuracy, training and tuning times. The suitability and superior performance of linear SVM is also experimentally analyzed, to conclude that this data acquisition technique generates linearly separable datasets.

## 1. Introduction

Over the last decade, the exponential growth of wind farms around the world has involved significant challenges, in order to improve their efficiency [1] and to reduce their operational costs [2]. The reduction of maintenance costs is the main strategy to reduce operational costs, because wind turbine maintenance is always a complex and expensive task, due to difficulties over their positioning and their distance from industrial areas. The recent development of offshore wind farms adds a further dimension to this industrial issue. The development and integration of on-line intelligent multisensor systems for the monitoring and diagnosis of wind turbines appears to be the best strategy to reduce their maintenance costs. However, an intelligent system, able to cover all possible failures, from electrical to mechanical, is still too great a problem to grapple with nowadays. For this reason, any monitoring system of wind turbines should focus on a wind turbine sub-assembly: the mechanical chain, the power generator, the lubricating system, *etc.* Once the sub-assembly is chosen, some steps should be followed: first, the most suitable sensors and their position in the sub-assembly should be chosen; second, the most suitable data-processing techniques should be found for each kind of signal collected from the sensors; third the best data-mining technique to extract as much information as possible from the processed signals should be identified. This Introduction addresses all these questions for a specific wind-turbine maintenance problem: the mechanical chain.

A variety of reviews on wind farm maintenance have been published [2–4]. Joselin *et al.* [2] include the power chain or gearbox and the rotor blade in the list of the more complex components in the performance of a wind turbine, and Tavner *et al.* [5] present these two elements as responsible for a significant number of wind turbine failures. The most common failures of these components are rotor blade misalignment and imbalance of the power chain due to bearing fatigue and gear damage [2]. Furthermore, the downtime due to such failures is even more important, due to procurement times and the need to winch these heavy sub-assemblies in and out of the nacelle.

There are different kinds of sensors that could be used for fault diagnosis of the gearbox and the generator subassemblies in wind turbines. Analysis of the power signals emitted by wind turbines is insufficient for this diagnosis. The limitations for the use of power signals in the mechanical diagnosis have their origin in the low-pass filter nature of the electrical motor itself. These power signals can be useful to detect concrete defects (of low frequency) in the generator, such as imbalance, but fail to detect the early stage of others defects, such as bearings (at first, of high frequency). In addition to that, due to the mechanical coupling between the generator and the rest of the power-train (gearbox and main shaft), the diagnosis of defects in those elements via power signals is even more difficult, if not impossible. Therefore, most of the sensors used for fault diagnosis in these devices attempt to evaluate vibrations generated during the rotation of these components. Accelerometers are therefore the most common sensors for this task. Vibration analysis has been studied and applied to rotating machinery for decades. It is widely accepted as one of the main fault diagnosis techniques in machine maintenance [6]. However, the case of wind turbines presents a new challenge in comparison with other rotatory machinery [7,8]: these devices work under variable conditions of load and speed. Only a few machines, such as excavators and helicopters, present these characteristics [9]. Therefore, the information obtained from accelerometers should be completed with information from other sensors that monitor the real-time rotating speed or the efficiency of the energy generation process, to locate the origin of the failure, and a multisensor approach is often proposed in the bibliography as a better solution to these industrial tasks [4].

Once the most suitable sensors have been selected, different approaches to data analysis may be chosen for failure detection in wind turbines. Spectral analysis of the power output signal has been proposed to monitor rotor blade imbalance [10] and gearbox and bearing faults [11]. However, in general, vibration sensors have been favored [12]. At first, the statistical features of the vibration signal in the time domain were studied [13], although this field of study quickly spread to include spectral analysis and time-frequency analysis [14].

Once signals from the sensors have been acquired, different techniques can be used to extract as much information as possible from these data, so as to build-up a suitable decision-making system for failure detection in wind turbines. Previous studies have applied different data-mining techniques to this industrial task, such as SVM [15,16], Bayesian networks [17], self-organizing maps [18], ANNs [19], ensemble classifiers [20] and neuro-fuzzy inference systems [16]. Moreover, recent works have proven the suitability of AI techniques for other similar industrial tasks, such as evaluation of the mechanical properties in rapid prototyping using ensembles [21], tool condition monitoring using *ν*-SVM [22] and ensembles of SVMs, hidden Markov model and the radius basis function [23].

SVMs with different kernels were tested on the dataset, to identify the quickest and the most reliable and accurate technique for this industrial task. ANNs were also tested as a standard technique to compare the results, as described in the Bibliography [13]. However, the use of ANNs has some disadvantages, as their results are highly dependent on the optimization of their tuning parameters, and the process of fine-tuning these parameters requires a lot of work and experience [24]. There are no general rules that may be followed as a guide, turning the scientific process into somewhat of an art. In contrast, SVM techniques are easier to use and have been successfully applied to many classification problems, such as recognition of isolated digits [25], objects [26], faces [27], speaker identification [28] and text categorization [29].

**Figure 1.**
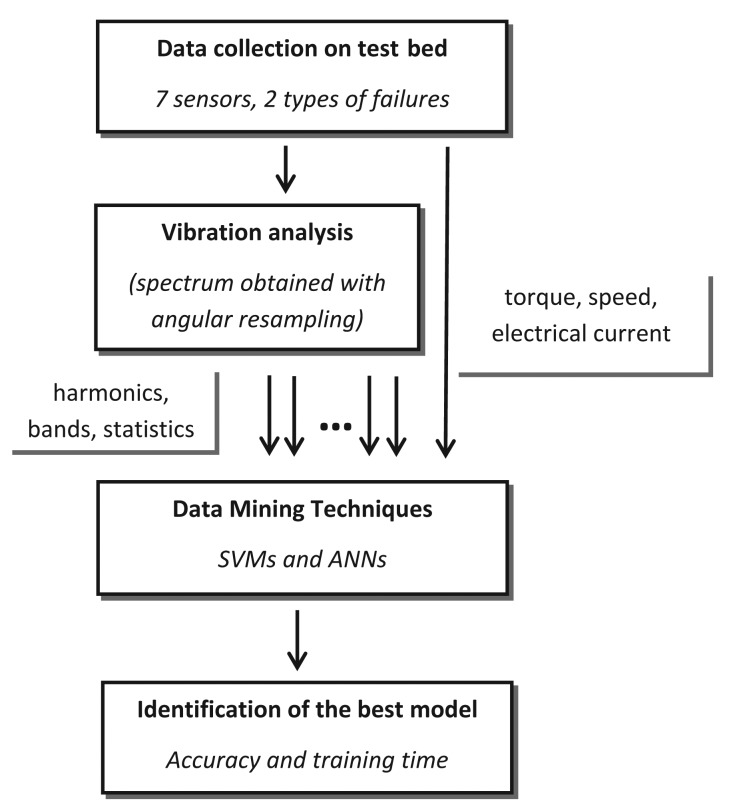
Diagram of the proposed methodology.

Even though extensive research has been completed into fault diagnosis in wind turbines, the standard technique used nowadays for this industrial task under real working conditions is the identification of critical variables by an expert and the development of a regression model that predicts the failure [14]. This solution requires in-depth knowledge of the mechanical chain and its components in such devices, especially to identify critical frequencies to detect imbalance failures. It has been adopted because existing studies are not suitable for real industrial conditions, as they follow two approaches with severe limitations. The first approach chose accelerometers as sensors and high frequency acquisition rates (kHz) to diagnosis the wind turbines. In this case, however, the recorded periods of time (in the order of months) are not enough to provide a sufficiently accurate failure map. Neither do these studies cover the wide working ranges of a wind turbine under real wind regimes, which necessarily involve operating conditions with very different speeds and loads. The second approach is found in SCADA systems integrated in the controls of real wind farms; these acquisition systems have no accelerometers, because their acquisition rates are low (Hz), and their predictive capabilities are therefore very limited. The novelty of this work is combining an extensive range of working conditions, obtained from a test-bed, with different failure levels for two of the most common failures in the mechanical chain of a wind turbine. In addition to this novel aspect, our study proposes a holistic strategy to build an optimized decision-making scheme to detect failures in such devices; Figure 1. Firstly, different sensors, including accelerometers, are setup in the wind turbine simulator, in positions that are also attainable in real wind turbines, and an extensive experimental program under random conditions is performed; secondly, a suitable technique for data processing of vibration signals at various rotation speeds is implemented; thirdly, different data-mining techniques, SVMs with different kernels, are tested on the dataset to identify the quickest, most accurate and reliable technique for this industrial task. ANNs were also tested, as they are a standard technique in the Bibliography, to compare the results [13]. This methodology also presents a further advantage regarding its implementation in real wind turbines; its configuration neither requires information on the mechanical design, nor on the presence of an expert, because the entire frequency spectrum is systematically analyzed (considering theoretical vibrational levels, to define all possible characteristic frequencies where faults may generate vibrations). The result of that analysis is a dataset containing sufficient representative instances for normal and failure working states. These instances also have a large number of features that help the machine learning method to choose the most suitable one for each wind turbine design. A very simple machine learning method, such as a linear SVM, with this specific dataset structure, outperforms some other sophisticated alternatives, as shown in the experiments. Similar schemes have been successfully applied to other industrial tasks, such as breakage detection [30,31] and roughness prediction [32]. In a previous study, we analyzed this dataset using ensemble classifiers [20], as this technique has been successfully applied to other similar industrial problems [24,32]. However, in this new work, we have obtained better statistically-significant results and a more reliable diagnosis system.

The paper is organized as follows. Section 2 introduces the experimental procedure for data collection, including a description of the test-bed, the sensors positioned on it and the failures that are generated, in order to simulate the real operating conditions of a wind turbine. Section 3 presents the analysis of the vibration signals obtained from the accelerometers and the final variables defined to generate the dataset. Section 4 presents the data-mining techniques used on the dataset for the classification task: SVMs and ANNs. Section 5 presents a detailed discussion of the results for these two methods in terms of accuracy, training and tuning times. Finally, Section 6 sets out the conclusions and future lines of work.

## 2. Experimental Procedure and Data Collection

**Figure 2.**
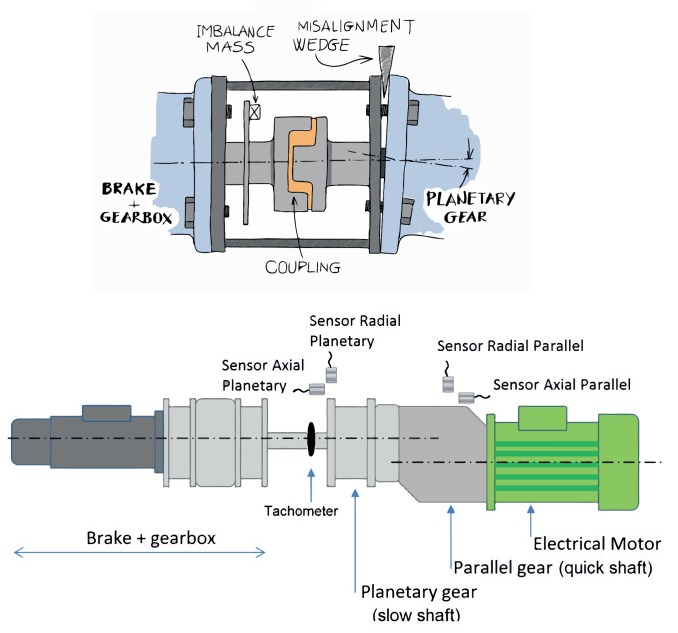
Scheme of the test-bed.

The experiments presented in this paper have been designed to simulate the behavior of wind turbines (variable speed and load) on a test-bed, in which defective operation can be simulated in a controlled way. The test-bed is composed of two sub-assemblies (Figure 2) that are separated by a mounting flange specially designed to maintain the alignment of the subassemblies. The first sub-assembly on the right of the picture includes a quick speed shaft, with an electrical drive (rather than a generator), a parallel gearbox and a planetary gearbox with a slow speed shaft. This set of gears (parallel + planetary) represents the configuration and the gear ratio of a commercial wind turbine of around 1:61. The left sub-assembly, composed by a brake and a two-stage planetary gearbox, simulates the wind conditions with a random variation of rotating speed and load. These two parts are connected through a specially designed steel structure that can control alignment and misalignment on both sides, thereby generating one of the failure mechanisms. A complete list of the mechanical characteristics of the test-bed has been presented in a previous work [33].

The vibrations were collected by four ICPaccelerometers in axial and radial directions from both gearboxes (parallel and planetary). The current and the torque of the electrical drive were also measured, and the speed of the slow shaft was registered by means of a laser sensor on a disk, using the same configuration that can be found in a real wind turbine for the measurement of low shaft speeds. In total, a 7-sensor system was developed for on-line monitoring of the test-bed performance. Figure 2 shows the positioning of all of the sensors.

The failures simulated on the test-bed were imbalance and misalignment, starting with small levels and increasing with each new set of measurements to simulate a progressive failure. Imbalance failure was simulated by attaching different masses to a disk used for speed measurement (for the imbalance), whereas misalignment failure was simulated by inserting a metallic wedge between the steel structure and the planetary gear on the right side of the test-bed (Figure 2). Altogether, seven failure cases and one non-failure case were tested. Table 1 shows the imbalance weight for each imbalance failure case, together with the equivalent percentage of the total weight of the rotor test-bed and the angle of each misalignment failure. The two levels of misalignment and the four levels of imbalance are enough to describe a progressive degradation of the mechanical chain of a wind turbine. Only one combination of both failures was tested, because this failure typology is very uncommon in real wind farms.

**Figure 3.**
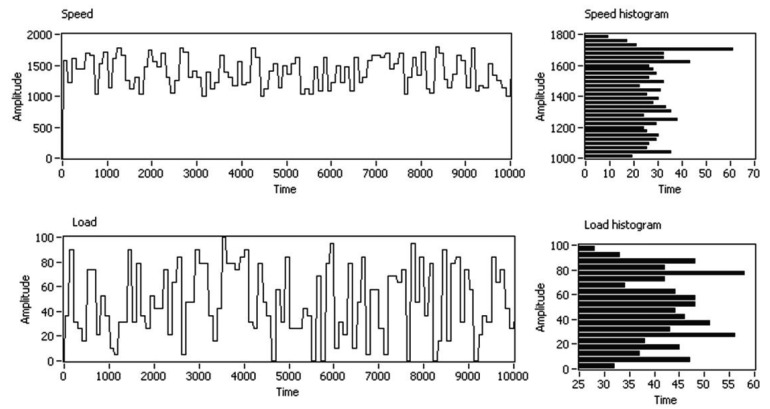
Example of random profiles of speed and load applied to the test-bed.

Random profiles of speed and load were programmed on the test-bed to cover an extensive range of real working conditions (e.g., a certain failure, once it appears, will be working under the load and speed conditions defined by the wind that is blowing in real time). Every 100 s, load and speed were stepped up to the next random condition, to generate a random profile of working conditions. The start of the speed test interval was chosen as 1000 rpm, because this is approximately the equivalent speed at which the slow shaft of a wind turbine begins to generate energy. The selected speed range was between 1000 and 1800 rpm, and the load range was between 0% and 100%. An example of these profiles of load and speed is shown in Figure 3, where the Y-axis shows the programmed amplitude of the speed and load for each experiment and the X-axis shows the time domain with a new step in load and speed conditions every 100 s. On average, each working condition was programmed on the test-bed and acquired 100 times by the multi-sensory system, in the whole measurement stage.

The measurement time to capture the vibration signal for each of the four accelerometers was fixed at 72 s (this gives an equivalent frequency precision of 0.014 Hz) with a sampling frequency of 25,600 Hz. The rotating speed was also measured on the slow shaft (Figure 2); this signal is used for angular resampling of the vibration signals, in line with the technique presented in an earlier work [34]. Each of these 72-s measurements generated an instance of the final dataset, following the procedure that is described in Section 3. In total, 6551 different working conditions were registered (considering failure level, load and speed), and so, the dataset will be composed of 6551 different instances.

## 3. Vibration Analysis and Variables Definition

Having collected the multi-sensory information from the tests, it was necessary to filter out the variations in speed and load included in each vibration measurement. This variation was artificially created during the tests to simulate real conditions in wind turbines: wind speed and force is not stable and fluctuates over time. However, the information that vibration sensors could provide, if this variation were presented, is very limited. The angular resampling technique makes it possible to eliminate speed variability.

Once this technique had been applied to the raw data of the accelerometer signals, in line with the procedure described in a previous work [34], different variables were calculated for each measurement interval of 72 s. These variables are mainly obtained from the analysis of the vibration spectrum recorded by the accelerometers. Some variables take account of the energy distribution; others take account of the energy allocated in the harmonics of the rotating speed and in standard frequency bands (in this case, the frequency spectrum is split into bands at a fixed position unrelated to the natural frequency of the system). It should be remarked that such a methodology is independent of the critical frequencies of the mechanical components of the test bed, and therefore, the system needs no expert knowledge for failure detection. The calculated variables, summarized in Table 2, are:

- Global variables associated with the operational state of the test-bed: torque, speed, electric input current (3 variables) and electric output current (4 variables).
- Energy contained in certain frequency bands associated with the potential mechanical faults that can occur in the bearings and the gears of the test-bed for each vibration signal (272 variables for all four accelerometer).
- Energy contained in certain order bands associated with possible mechanical faults in the bearings and the gears of the test-bed for each vibration signal (245 variables for all four accelerometers).
- Global statistical variables for each vibration signal: average, RMS, skewness, kurtosis and interquartile range (20 variables, 5 variables for each accelerometer).

Table 2 summarizes the final number of variables, which is 544, and the variation range of all of the variables.

The selected frequency bands and order bands have been presented in detail in a previous paper [33]. Some of the bands represent the characteristic frequencies of the different mechanical faults that may arise in machinery, such as imbalance, misalignment and gear faults. The calculation of these frequencies is well known in the literature and depends in many cases on geometrical parameters, such as the number of teeth of each gear (in our case, different gear ratios can be tested to define these bands, if the number of teeth is unknown). Other selected frequencies are the natural frequencies of the structure. These frequencies were selected in run-up test experiments, in which the rotation speed was increased with a ramp profile. After the experiments, the vibration was analyzed in the time-frequency domain, and the natural frequencies (constant) were separated from those mechanical frequencies that correlated with the speed (variable). This procedure may also be found in the literature [33].

**Figure 4.**
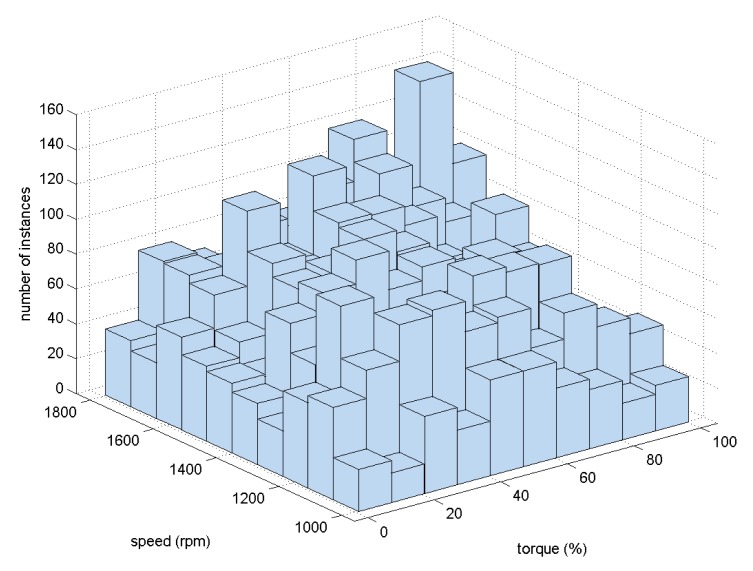
Histogram of the pair (torque, speed).

As has been explained in Section 2, 6551 different conditions were tested. The distribution of the instances among the fault cases is summarized in Table 3. From this table, it can clearly be concluded that the dataset was evenly balanced between the seven fault cases and the non-fault case. This balanced distribution of the instances is not common in such an industrial problem, because usually, the instances that describe failures and, specially, severe failures are very rare. The use of a test-bed in this research work is therefore clearly advantageous. It may be verified from Figure 4 that the random profile program generated an extensive map of different conditions. This figure shows the distribution of instances between the different speed (rpm of the slow shaft) and load (% of torque). Although the 6551 instances do not offer a completely homogeneous profile of the working conditions, all of the ranges are presented in the dataset with a significant number of instances.

## 4. Data-Mining Techniques

Once the dataset is generated, different data-mining techniques could be applied to it, searching for the extraction of as much information as possible about the behavior of the test bed under failure conditions. This section presents the data mining techniques applied to the dataset. First, an explanation is given of the two classification techniques that are tested; then, a discussion on feature-selection techniques is included, due to the huge amount of variables presented in the dataset. The two classification techniques used are ANNs, because they are the reference method for modeling this type of industrial problem [13], and SVMs, because of their characteristics that can outperform ANNs in this sort of industrial task.

### 4.1. Neural Networks

This classifier method is one of the most popular data-mining techniques. As indicated in the Introduction, it is probably the most frequently used in the context of fault diagnosis in rotating k-machines [13]. The extended use of this classifier could be due to two main reasons: first, it is a universal approximator of functions [35]; and, second, its mathematical formulation is inspired by biological functions, as it aims to emulate the behavior of a set of neurons. To do so, groups of small units, called neurons, are connected according to different architectures, the most habitual being the multi-layer perceptron (MLP). In the context of classification, this network has three layers, one with the network inputs (features of the dataset), a hidden layer and an output layer where the class is assigned to each input instance. The number of neurons in the input and output layer corresponds to the dimensions of the feature vector and the number of classes, respectively, but the number of neurons in the hidden layer is an important parameter of the structure of the ANN that needs to be adjusted.

When an instance is classified, firstly the output of the hidden layer, *y_hide_*, is calculated from the inputs, and then, the output, *y_hide_*, is taken as the input, according to the expressions shown in the formula [36]:

(1) $$\begin{array}{l} y_{\text{hide}}=f_{\text{net}}\left(W_{1}x+B_{1}\right)\\ y_{\text{output}}=f_{\text{output}}\left(W_{2}y_{\text{hide}}+B_{2}\right) \end{array}$$

where *W*_1_ is the weight matrix of the hidden layer, *B*_1_ is the bias of the hidden layer, *W*_2_ is the weight matrix of the output layer (e.g., the identity matrix in the configuration tested), *B*_2_ is the bias of the output layer, *f_net_* is the activation function of the hidden layer and *f_output_* is the activation function of the output layer. These two functions depend on the chosen structure, but are typically the identity for the hidden layer and *tansig* for the output layer [36]. It is possible to include the bias in the weight matrix, by considering an additional fixed input *x*_0_ = 1 and an output *y*_0_ = 1. With this change and substituting the expression of *y_hide_* into *y_output_*, it is reformulated as follows:

(2) $$y_{\text{output}}=f_{\text{output}}\left(W_{1}\times \left[f_{\text{net}}\left(W_{2}x_{\text{extended}}\right)\right]\right)$$

The back-propagation algorithm is used to obtain the weights *W*_1_ and *W*_2_ in these ANNs. This algorithm arrives at an initial estimate of the weights and then performs an iterative process that updates the weights according to the error between the predicted output and the real output. There are two important parameters in the learning process: the learning rate, *α*, and the momentum, *β*. Calling *t* the iteration number and *E* the error function, for each individual element of the matrix *W*_1_, *w_ij_*, the updating rule may be outlined as follows:

(3) $$w_{ij}\left(t+1\right)=w_{ij}(t)+\alpha \times \frac{\partial E}{\partial w_{ij}}+\beta \times \left(w_{ij}(t)-w_{ij}\left(t-1\right)\right)$$

Besides these two, a further parameter known as “epochs” can be used to limit the training time, stopping the loop for learning after a set number of iterations.

### 4.2. Support Vector Machines

The SVM technique [37] searches boundaries with the maximum margin of separation from the training data mapped into a space (called a space kernel) obtained from a transformation function. Margin maximization usually reduces the generalization error (*i.e.*, the expected error on a test set independent from the dataset used to build the classifier). We start the explanation of this classification technique with a geometrical view of the method, followed by its mathematical formulation. Firstly, the specific case of no data transformation is detailed (the case in which the kernel, as explained in subsequent sections, is linear, where the space kernel is equivalent to the original space) and, then, the general mathematical formulation and the types of kernels in use.

**Figure 5.**
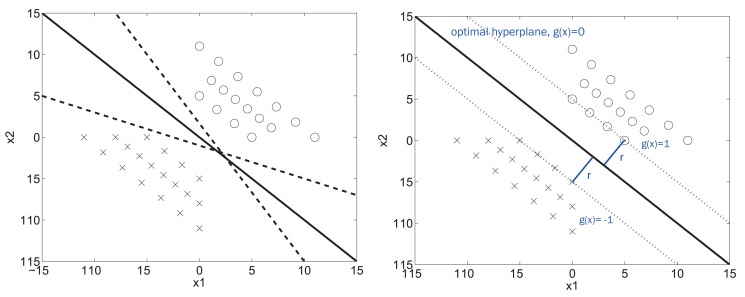
Idea of the SVM in the linear case.

Beginning with the ideal case of a linearly-separable dataset, a simple geometrical view of the idea behind the SVM, without mapping the data onto another space, is possible, as is the concept of the margin. In the left part of Figure 5, we have a class with its instances in crosses and another class marked in circles. The three lines that are drawn would be a valid way of separating the training dataset, but the continuous one appears to be a more appropriate way. Mathematically, if we define the margin as the distance between the boundary and the nearest parallel line that crosses an instance of any of the classes, the boundary that is drawn represents the boundary with the maximum margin (see the right part of Figure 5).

**Figure 6.**
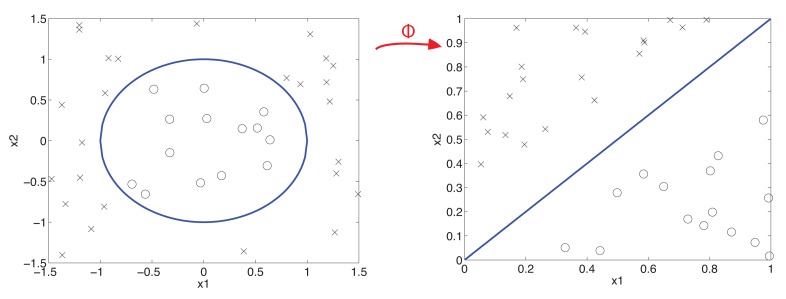
Graphical view of the SVM.

In practice, there are a lot of problems that mean the data may not be linearly separated, which is why a search is made for boundaries with a more complex geometry. In fact, in the case of SVM, a transformed space of the data is used, in which a search for the hyperplane is performed, as we can see in Figure 6.

In this transformation, a function (*ϕ*) is applied that searches the projection for linearly-separable areas. This can produce a space with more dimensions than the input space and even an infinite number (a case of infinite kernel techniques).

We start the mathematical formulation with the linear case, given a training set ${\left\{\left(x_{i},y_{i}\right)\right\}}_{i=1}^{N}$, which contains *D*-dimensional input vectors *x* ∈ ℜ*^D^* and their corresponding labels *y* ∈ {−1,+1}. The hyperplane that we want to find by maximizing the margin between classes is expressed as *g*(*x*) = *w^T^x* + *b*. The optimization problem can be written in terms of two parallel hyperplanes (*g*(*x*) = 1 and *g*(*x*) = −1, as shown in Figure 5):

(4) $$\begin{array}{l} \begin{array}{l} g(x)>=1,\text{if}\;y=1\\ g(x)<=-1,\text{if}\;y=-1 \end{array} \end{array}$$

The margin of separation between the parallel hyperplanes is 2/‖*w*‖. Maximizing the margin is equivalent to minimizing the inverse expression, 1/2‖*w*‖. The formulation of the optimization problem is shown in Equation (5), where ‖*w*‖^2^ is used instead of ‖*w*‖ for the convenience of carrying out the calculation process. The conditions *g*(*x*) >= 1, *if y* = 1 and *g*(*x*) >= −1, *if y* = − 1 can be expressed in one single equation, by taking the product *g*(*x*)*y*. This grouping gives us the formulation of the linear case, as follows:

(5) $$\begin{array}{l} \min_{w,b}y\frac{1}{2}{\left\|w\right\|}^{2}\\ \mathrm{s.t.}\;y_{i}\left(w^{T}x_{i}+b\right)>=1,i=1,\ldots ,n \end{array}$$

The previous scenario matches an idealized situation, and normally, the type of problem that is considered is non-linearly separable; a case in which the optimization problem that is formulated would have no solution. Therefore, the previous problem is generalized by means of a slack variable, *ε*, and a regularization parameter, *C*. The general formulation for the linear kernel is in this case:

(6) $$\begin{array}{l} \min_{w,b}y\frac{1}{2}{\left\|w\right\|}^{2}+C\sum_{i=1}^{n}\epsilon_{i}\\ \mathrm{s.t.}\;y_{i}\left(w^{T}x_{i}+b\right)>=1-\epsilon_{i},\epsilon_{i}>0,i=1,\ldots ,n \end{array}$$

where *ε* is related to the degree of error that is permitted, while the term *C* controls the importance that is attributed to the second term included in the minimization objective, to allow an error margin (the null parameter would imply no error).

Having seen the formulation for the linear case, the general SVM formulation will be presented. In general, the hyperplanes used as a decision boundary can not be formulated directly in the original feature space. Instead, a non-linear transformation of the data is completed beforehand, by using the “kernel trick”. The final expressions of the SVM optimization problem are detailed here. The hyperplanes are given by the the inner product (denoted by <.,.>) using a function *ϕ* that leads to the kernel transformation:

(7) $$\begin{array}{l} \min_{w,b,\epsilon }y\frac{1}{2}<w,w{>}^{2}+C\sum_{i=1}^{n}\epsilon_{i}\\ \mathrm{s.t.}\;y_{i}\left(<w,\phi_{x}>+b\right)>=1-\epsilon_{i},\epsilon_{i}>0,i=1,\ldots ,n \end{array}$$

In this case, the classifier is built as follows:

(8) $$g(x)=\text{sign}\left(<w,\phi_{x}>+b\right)$$

As the space in which the inner products are calculated can have infinite dimensions, the dual form of the problem is used by the SVM in its solution:

(9) $$\begin{array}{l} \min_{\lambda }\sum_{i=1}^{N}\sum_{j=1}^{N}\lambda_{i}\lambda_{j}y_{i}y_{j}K\left(x_{i},x_{j}\right)-\sum_{i=1}^{N}\lambda_{i}\\ s.t.\sum_{i=1}^{N}y_{i}\lambda_{i}=0,0<=\lambda_{i}<=C \end{array}$$

where *K* is the kernel function, defined for two different feature vectors *x_i_* and *x_j_* as *K*(*x_i_,x_j_*) =< *ϕ_x_i__*, *ϕ_x_j__* >, and the new optimizing objective is written in terms of the Lagrange multipliers, λ. Thus, the kernel function allows us to calculate the inner product between the mapped vectors without explicitly computing the mapping.

As an example of a kernel, we propose the radial basis kernel (or Gaussian kernel), given by a Gaussian distribution, with standard deviation parameter *σ*:

(10) $$K\left(x_{i},x_{j}\right)=\text{exp}\left(-\frac{{\left\|x_{i}-x_{j}\right\|}^{2}}{2\sigma^{2}}\right)$$

This type of kernel has two parameters that need to be optimized, the regularization parameter *C* and *σ*.

In this work, we have used four kernels, on the one hand, the two most commonly used—linear and Gaussian—and, on the other hand, two techniques with more novel kernels—stump and perceptron. These two kernels for infinite ensemble learning that are proposed in [38] were inspired by ensemble classifiers, combining good experimental results reached with both SVM and ensemble learning techniques. The aforementioned article explains how the kernel of an SVM should be generated, so that it is equivalent to an ensemble of infinite size. An ensemble is a combination of classifiers, usually trained with the same algorithm, but using different variations from the original dataset (e.g., subsampling, projections, instances reweighting, *etc.*) [39]. The ensemble prediction is usually computed by some weighted or non-weighted voting schema of the prediction of its members. Ensembles can improve their predictions by growing the number of their classifier members, so that is the aim of having a classifier, which theoretically emulates an ensemble of infinite size. Stump kernel is equivalent to an ensemble of infinite decision stumps. A decision stump is a decision tree with only one node. Therefore, it is a very simple classifier, as it makes its predictions by comparing only one feature of the instance against a precomputed threshold. Given that *x_i_* and *x_j_* are two instances, the kernel function *K*(*x_i_, x_j_*) for the stump kernel can be computed by summing the absolute values of the differences ‖*x_i_* − *x_j_*‖ for all of the dimensions of the original data (*i.e., l*1 norm). The perceptron kernel is equivalent to an ensemble of infinite perceptrons. A perceptron is perhaps the simplest ANN. It classifies instances by using a hyperplane in the original problem space as a decision boundary. *K*(*x_i_, x_j_*) for the perceptron kernel can also be easily computed by the Euclidean distance between both instances (*i.e., l*2 norm). Note that both the stump and the perceptron kernels have only one parameter to tune C, which is the common parameter for any SVM.

Finally, in [38], similar accuracy obtained by these kernels and the Gaussian kernel for real non-linearly separable cases is shown, but with less computational time, as it is necessary to adjust only one parameter, instead of two in the Gaussian case.

### 4.3. Variable Selection

The dataset includes 544 variables and 6551 instances. Such a large number of variables in comparison with the number of instances is due to the absence of an expert to select the most reliable variables to diagnosis failures in wind turbines. It is the only way to assure that the proposed intelligent system could be suitable for different mechanical chains without needing expert help for its configuration. Even though, from the mechanical and vibrational point of view, the selected set of variables that have previously been discussed are suitable for diagnosis misalignment and imbalance [33], this paper also presents a new analysis to confirm this conclusion, this time from a data-mining point of view. We used two variable selection techniques, in order to ensure that it was not possible to obtain classifiers with the same accuracy using fewer variables.

The variable or feature selection techniques are composed of two elements: a variable subset search method and a system of evaluation of their accuracy [40]. The second element, the accuracy evaluation system, allows us to group the feature selection techniques into two families [41]. The first one includes all techniques that use a filter variable selection technique, when the classification technique that will be used after the variable selection is not taken into account, but the attributes are chosen by means of statistical techniques that analyze the correlation that the attributes have between themselves and the class to be classified. The second group includes all techniques that use the classifier to evaluate the accuracy; in this case, there is a wrapper variable selection technique.

For this research, we have used one feature selection technique of each group. In the case of “filter type”, one of the most common variable selection techniques was used: “correlation-based feature subset selection”. In this method, the preference is for subsets of features that are highly correlated with the class, while having low intercorrelations. For the second group, an SVM classifier was used as an evaluator for the “wrapper” selection.

## 5. Results and Discussion

This section presents the results of variable selection and the performance of ANNs and SVMs in the dataset under study. The experiments were completed with WEKA [42] with an Intel Xeon E5405 (2 GHz) processor. Cross-validation was selected for both the training and the validation process, to assure that the results are independent of the instances selected for the training phase.

The ANN implementation of WEKAthat was used in the experimentation has three main parameters: one related to the structure of the classifier, the number of neurons of the hidden layer, and two related to the training phase, the momentum and the learning rate. Tuning of these three parameters of ANNs is a hard task if the dataset presents a large number of instances. An expert is needed to reduce this tuning time. For example, an automatic process to tune the ANN configuration of the three parameters was run on the dataset, without achieving an optimized result within a reasonable period of time. Specifically, using the WEKA “CVParameterSelection”tuning parameters method to adjust the three parameters at the same time, no repetition of a 5 × 2 cross-validation could be completed after five days of execution with the Intel Xeon E5405 processor. For this reason, the optimization process was run on only two training parameters (momentum and learning rate) for each specific configuration of the structure of the ANN (number of neurons in the hidden layer). In this way, the number of configurations tested in the same repetition was drastically reduced, and the computer was able to finish the tuning process in a reasonable time. The optimization method used a grid search provided by WEKA, with a 5 × 2 cross-validation, for the tuning of the pair momentum-learning rate. This method has two steps; in the first step, a two-fold cross-validation is used to estimate an initial optimum pair of the grid; then, an iterative process is performed, to confirm that the combination found in the first step is the best one. To do so, the best combination found in the first step is used as a center point and compared with its adjacent points through a 10-fold cross-validation, changing the pair and repeating the process if a better solution is found. Following this method, the first tests with 0, 5, 10, 15, 20 and 30 neurons in the hidden layer were performed. Table 4 shows the accuracy of the best configuration of ANNs found in each case, expressed in percentages of correctly classified instances. This analysis shows that the peak of maximum accuracy was around 20 neurons, in bold in Table 4, and any increase over this number of neurons does not improve the accuracy.

Then, configurations of around 20 neurons (*i.e.*, N = 15, 16,… , 23) were tested, and the optimization grid was performed to find the best momentum and learning rate for each configuration. Table 5 shows the result of this execution: the best result, in bold, was obtained for 17 neurons in the hidden layer (97.47%). We therefore have to consider that the most suitable structure of the ANN for this dataset is a 17-neuron configuration.

After the identification of the best ANN for this dataset, the SVMs were optimized. Sequential minimal optimization [43] is the SVM implementation used by WEKA. The stump and perceptron kernel had to be implemented within WEKA. SVM classifiers with the four types of kernels described in Section 4 were tested over the dataset, with a 5 × 2 cross-validation, while optimizing the corresponding parameters for each repetition and fold. In the Gaussian case, the regularization parameter, as well as the gamma parameter were optimized, while in the other kernels, it was only necessary to consider the regularization parameter. The same WEKA grid method used for optimizing momentum and learning rate of ANNs was used to optimize the SVM with a Gaussian kernel, whereas the other SVMs were optimized using a single parameter selection method.

Table 6 summarizes the results obtained from the optimization of SVM on the dataset and, for comparison, the accuracy of the best ANN configuration. Table 6 presents not only the accuracy of each data-mining technique, but also the tuning and training times, because both parameters are critical in real cases where an expert is not available to speed up the optimization process or where the computer is programmed to do so in an industrial environment. We used the corrected resampled t-test provided by WEKA [44] with a 95% confidence level to compare the methods. The best method, in terms of all three variables (accuracy, tuning and training time) is the linear SVM. The cases that are significantly worse than this best method are indicated with an asterisk. The accuracy obtained by an SVM with linear kernels is also of a higher statistical significance than the results of a previous study of this dataset using ensembles (96.24%) [20].

Besides the overall accuracy, a detailed decomposition of the error between the classes is shown, by means of the confusion matrix for the linear SVM case (Table 7). That matrix highlights the following issues:

1. There was 0% error in discriminating imbalanced failures *vs.* misalignment failures and discriminating any of these failures against the combination of both of them.
2. Only rarely (0.02%) did the classifier identify no failures, but Imbalance 2. Furthermore, on very few occasions (0.02%), imbalances of two and four were not recognized by the system.
3. Almost all errors in the confusion matrix occurred in the categorization of imbalance levels. Specifically, most of the matrix errors appeared in the discrimination between Imbalance 3 vs. Imbalance 4 (*i.e.*, 0.48% and 0.76%, which constitute 90% of all the matrix errors). This fact can be explained by the weights used to simulate each imbalance failure. As shown in Table 1, Imbalance 2 was simulated by applying approximately twice the weight used in Imbalance 1, and Imbalance 3 was also simulated by approximately twice the weight of Imbalance 2. However, this criteria of 100% weight increases was no longer valid when simulating Imbalance 4, where the weight only increased by around 50% in relation to Imbalance 3.

Taking account of accuracy alone, the best ANN configuration performed in a similar way to both SVMs with linear kernels and SVMs with stump kernels (see Table 6). However, when training and tuning time is also considered, SVMs with linear kernels clearly appear to be the most suitable method for this dataset, with a performance of half of the tuning time of other SVMs and at least 25% better in training time. If we compare ANN versus SVM performance, we can conclude that all SVMs require significantly less training time (at least 20-times less) than an ANN. In relation to the tuning time, the linear SVM is 10-times quicker than the best ANN configuration. The big difference between training and tuning times is remarkable and could be reasonably explained. Although ANNs and SVM with RBFkernels need to adjust two parameters, SVMs using the other kernels (linear, stump and perceptron) only need to fit one parameter, and therefore, in large datasets, there is a clear difference in tuning time. In fact, adjusting the parameters of an ANN demands more time than is actually shown in Table 6, which shows the time once the optimal number of hidden layers is known.

Linear SVM is the only method from the experiments that provides hyperplanes in the dataset space as decision boundaries. All of the remaining tested methods compute more complex decision boundaries (*i.e.*, non-linear boundaries). The better accuracy of linear SVM therefore suggests that the dataset is linearly separable. The dataset was divided into 28 new datasets to test this hypothesis, each one containing only all of the instances from two of the eight classes (the problem has eight classes, so there are 28 possible combinations of two classes). For each one of these subsets, a linear SVM is computed using all of its instances. These SVMs are validated using the same data that were used for training. If 0% error is not reached for a dataset, the C parameter of the SVM is then increased, and the linear SVM is computed again. Increasing C narrows the margin by trying to include more instances (*i.e.*, those close to the border) in the right side of the hyperplane. Remember that C penalizes the error from misclassified instances in the optimization problem (see Equation (6)). The results of this experiment show that even in the most difficult binary problem (Imbalance 3 vs. Imbalance 4), it is always possible to find a C value leading to a hyperplane with 0% training error. We may therefore conclude that the dataset is linearly separable.

Finally, a feature selection analysis was performed to assure the suitability of the selected variables, in order to diagnosis the two kind of failures. Table 8 summarizes the results of this analysis. Accuracy is expressed by the percentage of correctly classified instances. The standard deviation is included between parentheses for each magnitude. As previously explained in Section 4, two feature selection techniques were tested: a filter selection and a wrapper selection. The accuracy of both techniques is never higher than 95.6%; but a linear SVM with the completed dataset is able to achieve a significantly higher accuracy of 98.0%, without any tuning of its parameter. It may be concluded that the original dataset cannot be reduced in terms of variables without losing information.

Feature selection results point out that all of the 544 features used by the hyperplanes that are computed with linear SVM are necessary to obtain significantly better results. Therefore, linear SVM performs better, because the dataset has been built using features that are very suitable for creating a linearly-separable dataset.

## 6. Conclusions and Futures Lines of Work

A promising architecture to detect bearing failures in wind-turbine gearboxes has been presented that comprises a combination of angular resampling for vibration analysis, monitoring of the wind-turbine power output and data-mining techniques for the classification task. To overcome the lack of datasets with a wide range of conditions of loads and speeds, different experiments have been performed on a test-bed for two common failure mechanisms: misalignment (two levels of failure) and imbalance (five levels of failure), in addition to the non-fault case. The whole dataset included 6551 instances with 544 variables, such that it can be considered a high dimensional problem. The dataset was mainly balanced within the seven fault cases and the non-fault case, the contribution of each one to the final data set being higher than 8.4% and lower than 13.5%.

SVMs are state-of-the-art classifiers that are more widely used in real problems because of their performance. In this paper, the most traditional kernels (*i.e.*, linear and Gaussian), besides some new kernel techniques that could be suitable for this industrial problem (*i.e.*, perceptron kernel and stump kernel) have been tested. The results have also been compared with ANNs, which are taken as a baseline method, as they are broadly used in other industrial classification problems. All parameters of the selected classifiers were tuned in the study. As the ANN tuning processes are time-consuming, instead of optimizing the number of the neurons'momentum learning rate in the output space, optimization of the momentum learning rate is performed for different values of the number of neurons.

The results showed that linear SVM performed best and is significantly better than other sophisticated kernels, such as the Gaussian kernel or the perceptron kernel. The stump kernel and ANNs were outperformed by linear SVM, with only slight differences, but only if accuracy is considered. The confusion matrix for linear SVM shows 90% of errors in discrimination between Imbalance 3 vs. Imbalance 4, but the other binary problems either have no error or they are smaller than 0.02%. The concentration of errors in this binary problem is also explained by the way they were simulated. However, the performance of these techniques has also been compared in terms of tuning and training times, because both parameters are critical in real industrial applications when the computer data cannot be interpreted by an expert prior to the optimization task. It is the simplicity of the linear SVM method that makes it quicker, with training times that are much shorter than the time required by the other algorithms that have been tested. It outperforms the other algorithms when tuning time is considered, because of its short training time with only one parameter to tune. In contrast, ANNs have more parameters to tune, and their training times are very high; thus, it takes much longer for an ANN to achieve competitive results in its classification performance. All of these reasons make linear SVM the most suitable classifier for the problem proposed in this study. The reason behind the better accuracy of linear SVM has also been explored. The experiments point out that a linearly-separable dataset is obtained using the proposed methodology.

Future work will focus on the application of this methodology to other types of wind-turbine failures, so that the same software solution can fully identify the performance of the wind turbines. Moreover, the application of this methodology to real data from different wind farms is foreseen, where there will be a clear imbalance in the dataset between non-failure cases and failure cases.

## Figures and Tables

**Table 1.** Type and level of faults.

**Imbalance**			**Misalignment**	
	
**Label**	**Weight (g)**	**%**	**Label**	**Angle (°)**
Imbalance 1	5.79	0.077	Misalignment 1	0.78
Imbalance 2	9.13	0.12	Misalignment 2	1.53
Imbalance 3	19.5	0.26		
Imbalance 4	28.8	0.38		

**Table 2.** Variables included in the final dataset.

**Operation State**			

**Magnitude**	**Number of Variables**	**Units**	**Range**
Torque	1	% of maximum torque	1–100
Speed	1	rpm	1000–1800
Input current	1	Amperes	1.63–2.85
Electrical current in the axis	4	Amperes	2 ×10^−4^–0.12

**Vibration Analysis**			

**Magnitude**	**Number of Variables**	**Units**	**Range**

Harmonics	272	10^−3^× mm/s^2^	5.63 ×10^−3^–0.075
Bands	245	10^−3^× mm/s^2^	1.65 ×10^−2^–0.052
Average	4	10^−3^× mm/s^2^	−2–4
RMS	4	mm/s^2^	0.016–0.12
Skewness	4	dimensionless	1.40–4.78
Kurtosis	4	dimensionless	2.19–66.1
Interquartile range	4	mm/s^2^	0.021–0.17

**Table 3.** Distribution of measurements between the different instances of failures that were tested.

**Type of Fault**	**Number of Instances**
No misalignment, No imbalance (NO)	887 (13.54%)
No misalignment, Imbalance 1 (IM1)	847 (12.93%)
No misalignment, Imbalance 2 (IM2)	856 (13.07%)
No misalignment, Imbalance 3 (IM3)	838 (12.79%)
No misalignment, Imbalance 4 (IM4)	864 (13.19%)
Misalignment 1, No imbalance (MI1)	872 (13.31%)
Misalignment 2 No imbalance (MI2)	835 (12.75%)
Misalignment 2, Imbalance 4 (IM4 + MI2)	552 (8.43%)

**Table 4.** Accuracy of the ANN changing neurons in the hidden layer.

**Hidden Layers**	0	5	10	15	**20**	30

**Accuracy**	95.79	96.66	97.15	97.20	**97.34**	96.96

**Table 5.** Accuracy for a number of neurons of around 20.

**Hidden Layers**	15	16	**17**	18	19	20	21	22	23

**Accuracy**	97.20	97.12	**97.47**	97.05	97.26	97.34	96.96	97.39	97.15

**Table 6.** Summary results of SVMs vs. ANNs.

	**Linear SVM**	**Perceptron SVM**	**Gaussian SVM**	**Stump SVM**	**ANNs**
**Accuracy**	**98.26**	96.86	97.25	**97.85**	**97.47**
**(%)**	(0.24)	(0.36) *	(0.31) *	(0.23)	(0.24)
**Tunning**	444.27	1,241.27	16,068.97	945.17	46,611.12
**time (s)**	(10.38)	(16.27) *	(35.42) *	(11.19) *	(2,391.96) *
**Training**	**12.48**	21.55	15.61	22.17	491.00
**time (s)**	(0.40)	(0.31) *	(0.28) *	(0.28) *	(10.77) *

* The method is significantly better

**Table 7.** Confusion matrix for the linear SVM classifier.

***Forecasted Class***									

		**NO**	**IM1**	**IM2**	**IM3**	**IM4**	**MI1**	**MI2**	**IM4 + MI2**
*Real class*	**NO**	13.52	0	0.02	0	0	0	0	0
	**IM1**	0.01	12.73	0	0.16	0.03	0	0	0
	**IM2**	0	0	13.02	0.04	0	0	0	0
	**IM3**	0	0.14	0.04	11.85	0.76	0	0	0
	**IM4**	0.01	0.04	0	0.48	12.66	0	0	0
	**MI1**	0	0	0	0	0	13.31	0	0
	**MI2**	0	0	0	0	0	0	12.75	0
	**IM4+MI2**	0	0	0	0	0	0	0	8.43

**Table 8.** Accuracy of a SVM classifier with variable selection.

**Filter Selection**	**Wrapper Selection**	**Default SVM**
95.57 (0.38)	95.17 (0.37)	98.02 (0.26)
